# The Differences in Acute Management of Asthma in Adults and Children

**DOI:** 10.3389/fped.2019.00064

**Published:** 2019-03-11

**Authors:** Richard Chavasse, Stephen Scott

**Affiliations:** ^1^Consultant Respiratory Paediatrician, St George's University Hospitals NHS Foundation Trust, St George's University of London, London, United Kingdom; ^2^Consultant in Respiratory Medicine, The Countess of Chester Hospital NHS Foundation Trust, Cheshire, United Kingdom; ^3^Chester Medical School, The University of Chester, Chester, United Kingdom

**Keywords:** asthma, attack, adults, children, treatment

## Abstract

Acute asthma or wheeze is a common presentation to emergency services for both adults and children. Although there are phenotypic differences between asthma syndromes, the management of acute symptoms follow similar lines. This article looks at the similarities and differences in approaches for children and adults. Some of these may be age dependent, such as the physiological parameters used to define the severity of the attack or the use of age appropriate inhaler devices. Other differences may reflect the availability of evidence. In other areas there is conflicting evidence between adult and pediatric studies such as a temporary increase in dose of inhaled corticosteroids during an acute attack. Overall there are more similarities than differences.

## Introduction

Asthma is the commonest chronic condition in the UK with a UK lifetime prevalence of patient-reported clinician-diagnosed asthma of 15.6%. In 1 year asthma results in 6.3 million primary care consultations and 93,000 hospital in-patient episodes. The costs of asthma are estimated at £1.1 billion 12% of which is accounted for by hospital care and 14% for primary care consultations (1). Many physicians in both adult and pediatric medicine across the whole healthcare economy will therefore be expected to manage patients who may present with an acute attack of their disease so knowledge of this area is very important for good patient care. Guidelines for the management of an acute asthma attacks are documented in a number of national and international publications (2–4). Management of an acute attack may start with treatment at home and may progress to treatment in primary care, the emergency department and on the hospital wards including intensive care. This article looks at similarities and differences in the management of acute management between adults and children of varying ages.

The definition of asthma is difficult and there is no gold standard, and it is increasingly recognized that asthma as a condition is made up of a number of phenotypes (5, 6). The use of phenotypic variations is more likely to inform and modify chronic management, rather than the acute management of a crisis at the present time. The first recognized presentation of asthma is not uncommonly an acute attack, particularly in children, and therefore may present a diagnostic challenge.

The British Thoracic Society and Scottish Intercollegiate Guidelines Network (BTS/SIGN) asthma guideline precis the definitions of asthma as the presence of more than one symptom of wheeze, breathlessness, chest tightness and cough associated with variable airflow obstruction. Other definitions of asthma, in both children and adults, include airway hyper-responsiveness and airway inflammation as components of the disease (BTS/SIGN) (2). The National Institute of Clinical Effectiveness (NICE) have suggested a diagnosis should not be made without objective evidence of airway obstruction or inflammation (3).

In younger children, particularly those <5 years of age, the diagnosis of asthma (or phenotypes of wheeze) is difficult and often controversial (7). Acute wheeze is however a very common presenting complaint to both primary and secondary care and may frequently be recurrent and troublesome. Whilst the intricacies of diagnosis is beyond the context of this piece, recognition of this difficulty is important. Asthma diagnosis and phenotypes are subjects covered elsewhere in this series of articles. As is not uncommon, the evidence for different treatments are less well studied in the younger age groups. For the purpose of this review it will be assumed that the diagnosis of asthma is secure and we will be comparing / contrasting the differences in children and adult asthma.

## Definition of Adults and Children

The definition of an adult will be taken as a person over 18 years, accepting there is a considerable cross over in the adolescent age group. In most hospital emergency departments, adolescents under 18 are usually managed in the pediatric area, and if necessary by pediatricians, although it is recognized that different healthcare systems may have different policies. Guidelines and clinical trials vary in their cut off ages with many studies including adolescents over 12 in “adult” studies. Age cut offs are clearly not as relevant to provision of care in primary care and the home environment, however the age and maturity of the individual patient should be considered when agreeing a management plan. For children there are differences in the approach to the young, preschool child (<5 years) and there is very little evidence for the management of asthma symptoms in children under 1 year of age.

## Definitions & Assessment of Acute Asthma

An acute asthma attack represents a deterioration in symptoms and lung function from the patient's normal status. Distinguishing between a lower respiratory tract infection and an asthma attack can be challenging, particularly in young children. This can include shortness of breath, wheezing, cough, and chest tightness (4). Attacks are marked by a decrease from baseline in objective measures of pulmonary function, such as peak expiratory flow rate and FEV1. Objective measures, such as FEV1 are less easy to perform in children and young persons, and PEFR can be unreliable in children and young persons if they have not used the technique before and are highly unlikely to be achieved in children <5 years of age (8). In some individuals, in particular those with poor adherence and/or more severe asthma, there can be a challenge in distinguishing between a mild asthma attack and long-standing poorly controlled asthma; the challenge in in part due to symptoms being common to both a mild asthma attack and poorly controlled asthma and in part due to guidelines not defining criteria for a mild asthma attack.

Pharmacological management is entirely dependent upon the severity of the attack which in turn is defined by a number of objective and subjective findings. These differ between adults and children, reflecting the age-related physiologic differences and the reliability of pulmonary function testing in children. Definitions from the BTS/SIGN and Global Initiative for Asthma (GINA) guidelines are outlined in Table 1.

**Table 1:** Comparison of symptoms and signs associated with levels of acute asthma severity in adults and children the BTS/SIGN (1) and GINA (4) asthma guidelines.

	**BTS/SIGN**		**GINA**	
	**Adult**	**Children**	**Adult & Children >5years**	**Children (0-5)**
Moderate	Increasing symptoms PEFR >50–75% best or predicted No “severe” symptoms	Able to talk in sentences SpO_2_ ≥ 92% HR-≤140/min^*^ or 125/min^**^ RR-≤40/min^*^ or 30/min^**^ PEFR ≥50%^**^ best or predicted No “severe” symptoms	Talks in phrases Prefers sitting to lying Not agitated Respiratory rate increased Accessory muscles not used Pulse rate 100-120 bpm SaO_2_ in air 90-95% PEF >50% predicted or best	Breathless Agitated Pulse rate ≤200 bpm (0–3), ≤180 (4–5 years) SaO_2_ ≥92%
Severe	Any one of: Inability to complete sentences in one breath PEFR: 33–50% best or predicted HR ≥ 110/min RR ≥ 25/min	Can't complete sentences in one breath or too breathless to talk or feed SpO_2_ <92% HR ≥ 140/min^*^ or 125/min^**^ RR ≥ 40/min^*^ or 30/min^**^ PEFR 33–50%^**^ best or predicted	Talks in words Sits hunched forwards Agitated Respiratory rate >30 bpm Accessory muscles in use Pulse rate >120 bpm SaO_2_ <90% in air PEFR ≤50% predicted or best	Unable to speak or drink Central cyanosis Confusion or drowsiness Marked subcostal and/or sub-glottic retractions SaO_2_ <92% Silent chest on auscultation Pulse rate >200 (0–3 years) or >180 bpm (4–5 years)
Life threatening	Severe attack with any of: Altered conscious level Exhaustion Arrhythmia Hypotension Cyanosis Silent Chest Poor respiratory effort PEFR <33% best or predicted SpO_2_ <92% PaO_2_ <8 kPa Normal PaCO_2_ 4.6–6 kPa	Severe attack with any of:Silent Chest Cyanosis Poor respiratory effort Hypotension Confusion Exhaustion SpO_2_ <92% PEFR <33%^**^ best or predicted	Drowsy Confused Silent chest	
Near fatal	Raised PaCO_2_ and/or requiring mechanical ventilation			

* children 1–5 years;

** *children over 5 years*.

## Pre-hospital Management of Acute Asthma

Adults are encouraged to have a personalized asthma action plan (PAAP) in place which empowers them to increase treatments in response to increasing symptoms or decreasing PEFR (9). The PAAP should advise them when to seek medical assistance. In children PAAPs advice only recommends increasing short acting beta agonist. A Cochrane database systematic review of 4 clinical trials in children concluded that symptom-based PAAPs are superior to peak flow PAAPs for preventing acute care visits (10). There was insufficient data to firmly conclude how symptom-based PAAPs were superior of the two. For example the observed superiority of symptom-based PAAPs may have been due to greater adherence to the monitoring strategy, earlier identification of onset of deteriorations, higher threshold for presentation to acute care settings, or the specific treatment recommendations (10).

Most adult PAAPs recommend the increased use short acting beta agonists at the onset of acute symptoms (11). Most pediatric management plans suggest a titrated increased dose of beta-2 agonist using doses between 2 and 10 puffs of a salbutamol (pressure dose metered dose inhaler and spacer device) up to every 4 h (12). Safety net advice to seek medical advice and call an ambulance if not responding to treatment should be included. Judicial use of rescue oral steroids at home is sometimes included, although there is no consistent evidence of benefit for this practice (13).

A second approach in adults may be the use of a single combination inhaler for both preventer and relief [Maintenance and Reliever Therapy: (MART)] which patients may titrate in accordance to their symptoms (14). Preparations are not currently licensed or in formulations for younger adolescents or children in the UK.

A recent study in adults suggested a 4-fold increase in inhaled steroid dose used early during an attack resulted in fewer severe asthma exacerbations than a plan in which the dose was not increased and this would be a reasonable self-management strategy in this situation (15). A similar pediatric paper suggesting a quintupled dose of ICS in children however showed no benefit (16). This may reflect a difference in causation of an attack, different responses by age or methodological differences between the studies.

Any patients with features of acute severe or life-threatening asthma should be referred to hospital immediately.

## Medical Management of Acute Asthma in Healthcare Facilities

### Oxygen

It is essential to monitor oxygen saturations when assessing an asthma attack. In adults there is evidence that hyperoxia may be detrimental plus there is also a possibility that the patient may have chronic obstruction so that the delivery of oxygen should be controlled to maintain an SpO2 level of 94–98% (17).

In children with saturations <92% in air after initial bronchodilator treatment it is advised that inpatient hospital treatment will be required as this reflects more severe asthma (18, 19). Oxygen should be administered to any child with acute asthma with SpO2 <94% via a tight fitting face mask or nasal cannula at sufficient flow rates to achieve normal saturations of 94–98%.

### Short Acting Bronchodilators

The initial management of an acute asthma attack is usually given by the inhaled route. In adults it is recommended that in non-life threatening acute asthma, beta agonists can be given through repeated actuations of a pMDI via an appropriate large volume spacer. The recommendation is to give 4 puffs (400 mcg) as the initial dose and increase by 2 puffs every 2 min up to 10 puffs if needed. In life threatening asthma however wet nebulization of beta agonists with oxygen is preferable. Adult patients who do not respond to initial nebulization of beta agonist may be considered for repeated doses at 15–30 min intervals or continuous nebulization at 5–10 mg/h (2).

In Children, a pMDI and age-appropriate spacer device is the preferred option for delivering inhaled beta agonists with mild to moderate asthma as this is less likely to produce a tachycardia and hypoxia than using a nebulizer (20, 21). A facemask connected to the mouthpiece of a spacer is recommended in children <3 years old. In children drug dosing should be escalated as required from two puffs of salbutamol suitable for mild attacks up to 10 puffs for more severe attacks with 30–60 s between puffs. The inhalers should be activated into the spacer in in individual puffs and inhaled immediately by tidal breathing (for five breaths). In the hospital setting, with appropriate monitoring, doses can be repeated every 20 min over an hour if necessary. In children with severe or life-threatening asthma should receive nebulized bronchodilators driven by oxygen. These can be given in combination with ipratropium bromide which is recommended if there is a poor response to salbutamol alone (22). Repeated doses can be given every 20 min over a 1–2 h period. In contrast to adult recommendations continuous nebulized beta agonists are of no greater benefit than the use of frequent intermittent doses in the same hourly dosage (2, 23, 24).

After the first few hours salbutamol can be tapered down to one to two hourly and ipratropium tapered to four to six hourly or discontinued. In adults ipratropium bromide is recommended in acute severe or life-threatening asthma but is not necessary in milder asthma attacks or after stabilization.

### Steroids

For adults and children it is important to give steroids early during an acute attack. Steroid tablets are as effective as injected steroids and should be given for at least 5 days in adults or until recovery. Evidence for the ideal duration of oral steroid treatment is still required (25). Once recovery is achieved, steroids can be stopped abruptly and do not need to be tapered unless the patient is on maintenance steroids or if they have received a prolonged course of three or more weeks (26). Inhaled steroid treatment should continue during prescription of oral steroids (2).

In children, oral prednisolone is the steroid of choice for asthma attacks and intravenous corticosteroids are only indicated for children who are vomiting or who have very severe symptoms which prevent swallowing (27). Some studies using dexamethasone have shown potential equivalence to prednisolone with the potential to reduce the number of doses and therefore potentially improving treatment adherence (28). The use of oral steroids is more controversial in preschool children (age 1–5). This may reflect the presence of common symptoms in asthma and lower respiratory tract infection in this age group. Additionally there may be more than one acute asthma phenotype, but one study considered this and found no difference in outcome between children with “viral induced wheeze” with and without atopy (29). Different prednisolone dose schedules have been recommended from 0.5–2 mg /kg (30) and others recommend an age related dose schedule (2). In children recommendations are usually for 3 days of treatment but may need to be extended from 5 to 10 days to bring about recovery. Tapering is not necessary unless the course exceeds 14 days.

There is no evidence to support the use of Inhaled steroids as an alternative to oral steroids during an asthma attack of this severity but it is good practice to continue the usual maintenance inhaled steroids during an attack (2).

### Second Line Treatments

#### Magnesium

There is some evidence that intravenous magnesium sulfate has bronchodilator effect during an acute asthma attack (31). In adults there is limited evidence that a single dose of iv magnesium may be some benefit in adults with a PEF <50%. This is felt to be safe and may improve lung function and reduce intubation rates in those with acute severe asthma. It may also reduce admission to hospital with asthma from ED in adults who have had little or no response to standard treatment. It should only be used following consultation with senior medical staff (2).

The use of IV magnesium has become more frequent in children with acute asthma. There is relatively little evidence compared to other intravenous therapies although there is one comparative study showing a more rapid improvement compared to IV salbutamol or aminophylline (32). It is generally safe with few side effects. It should be used if there is a poor response to first line therapies and is not recommended in mild to moderate asthma attacks.

The use of nebulized magnesium has been studied in one large UK based trial (33). Overall the benefits were small although did seem to offer some benefit when added to nebulized salbutamol and ipratropium in the first hour of hospital treatment in children presenting with a short duration of acute severe asthma symptoms presenting with an SpO2 <92% (34). At the time of writing, the role of nebulized magnesium in both adults and children is uncertain.

#### Aminophylline

In adults evidence suggests that the addition of intravenous aminophylline is unlikely to add any additional bronchodilation compared to standard care and side effects such as arrhythmias and vomiting are increased (35). However, patients with near fatal asthma with a poor response to initial therapy may gain additional benefit from IV aminophylline. It is advised that before its use consultation should take place with a senior member of medical staff as these patients are likely to be rare (2).

In children, aminophylline was previously considered the first line IV treatment following a poor response to initial management (bronchodilators and steroids) but now intravenous magnesium is recommended as first line due to reduced side effects and equal efficacy (2). There is some evidence of benefit in severe or life threatening asthma (36). The risk of side effects is high and ECG monitoring is recommended with the patient monitored in an HDU/PICU environment.

#### IV Salbutamol

There may be some benefit for the use of IV salbutamol in both adults and children who have not responded to first line treatments and IV magnesium (37).

In children an initial bolus dose (15 mcg/kg) may be given. In comparison with IV aminophylline (bolus and infusion) this has been shown to give equivalent results. Initially it was thought to cause fewer side effects compared to aminophylline but more recently nausea, tachycardia and lactic acidosis have been frequently recognized. There is very limited evidence for the use of a salbutamol infusion in children but is sometimes utilized in the PICU environment.

#### Antibiotics

In adults an infection precipitating an attack is most likely to be viral and therefore routine prescription of antibiotics is not indicated. Objective measures such as serum procalcitonin where available should be used to guide decisions on using antibiotics (2).

In children the role of bacterial infection is also less common than a viral trigger. Antibiotics are not recommended. Procalcitonin is not routinely used at present in children. CRP is more frequently used but less specific.

#### Critical Care

In both adults and children with acute asthma and a poor response to standard therapy it is important to involve clinicians with the appropriate skills in airway management and critical care support early. There is little good quality evidence to guide treatment at this stage and a national audit of treatment for acute asthma in adults in the UK found a wide variation in clinical practice (38).

In adults admission to critical care is recommended in those requiring ventilator support and acute severe life-threatening asthma indicated by: deteriorating PEF, persisting or worsening hypoxia, hypercapnia, arterial blood gas analysis showing fall in pH or rising hydrogen concentration, exhaustion, feeble respiration, drowsiness, confusion, altered conscious state or respiratory arrest (2).

In children admitted to hospital with status asthmaticus two small studies have reported the use of non-invasive ventilation suggesting that it is safe and feasible but there was insufficient evidence of its effectiveness. In adults there has also been suggestions that the use of NIV can be safe and effective however the evidence is limited and inconclusive. One trial of NIV in a small number of patients showed improvement in hospitalization rates, discharge from emergency departments and lung function. Other trials have shown safety and feasibility of using NIV in treating acute exacerbations of asthma but little evidence of benefit in comparison with standard care. It is recommended that NIV should only be considered in adults in a critical care or equivalent setting (2).

Invasive ventilation strategies are beyond the scope of this article.

The BTS asthma guidelines are able to recommend the following advice for therapies at this stage. In both adults and children Ketamine may be considered as a potential bronchodilator although further prospective trials are required before conclusions about effectiveness can be drawn (39, 40).

In children the anesthetic gas Sevoflurane is potentially an option for correcting high levels of PaCO2 in mechanically ventilated children however its use would be limited to areas where appropriate scavenging facilities to extract gas are available (41).

There is an increasing trend in the use of Recombinant DNase in pediatric intensive care in children with acute asthma due to airway plugging. There is little or no published evidence to support this practice. A study in non-intubated adults with severe asthma showed no improvement in FEV1 (42).

### Extracorporeal Membrane Oxygenation (ECMO)

ECMO is a form of cardiopulmonary life-support, where blood is drained from the vascular system, circulated outside the body by a mechanical pump, and then reinfused into the circulation. While outside the body, hemoglobin becomes fully saturated with oxygen and CO2 is removed. Oxygenation is determined by flow rate, and CO2 elimination can be controlled by adjusting the rate of countercurrent gas flow through the oxygenator. It is a highly specialized form of treatment and available in only a few centers in the UK. It has been shown to be useful and an option in severe asthma refractory to mechanical ventilation in both children and adults by providing adequate gas exchange and preventing lung injury. However, careful management is required to avoid complications (43).

### Discharge and Follow Up

In adults it is recommended discharge can be considered when clinical signs are compatible with home management i.e., medical therapy can continue safely at home. Patients with PEF <75% best or predicted and diurnal variability of PEF >25% are at greater risk of early relapse and readmission therefore this should be borne in mind when deciding on timing of discharge. This is also true in children (2).

In both adults and children it is important to assess and attend to correct risk factors that may have lead to a loss of control of asthma leading to admission e.g., smoking or exposure to smoke in the household in children. Education prior to discharge is important including advice on inhaler technique, a review of the personal asthma plan and, if used, PEF record keeping. Tools such as the BTS asthma discharge bundle are available to provide help with this process (44).

All patients both adult and children should have an appointment arranged with their primary care team within 48 h of hospital discharge. Secondary care review should be arranged within a few weeks.

## Conclusion/Summary

There are similarities and differences in the diagnoses, assessment and treatment of acute asthma in adults and children, and these are summarized in Table 2. The mainstay of management is to ensure early assessment of severity with appropriate use of bronchodilators with corticosteroids. There are some differences in the recommendations of how these medications are given and their associated doses but because the pathological processes are essentially the same the general principles of treatment are also the same. The more cautious use of oral corticosteroids in children may reflect concern over longer term side effects as well as questionable response in preschool children. The use of variable dose regimes of inhaled steroids may reflect the availability of suitable drug preparations and the difference between an individual feeling their own innate symptoms and a parent recognizing their child's symptoms.

**Table 2:** Summary of similarities and differences in the diagnoses, assessment and treatment of acute asthma in adults and children.

	**Similarities**	**Differences**
Diagnosis	Same symptoms	Challenge in distinguishing from lower respiratory tract infection may be greater in children
Assessment	The same physiological parameters are used in assessment (i.e., Respiratory rate, Heart rate, Oxygen saturations)	Physiological parameters differ numerically between adults and children. Achievability and reliability of PEFR and FEV_1_ in children is lower than in adults
Stepping up treatment	Actions plans recommend using short acting beta agonists when symptoms occur	Only adult personal action plans (based on good evidence) recommend MART of quadroupling ICS dose for worsening control/minor asthma attacks
Supplemental oxygen treatment	Required when oxygen saturation are below 92% in all age groups	Aim for ceiling 98% in adults due to potential overlap with COPD
First line treatment	Same medications used	Doses differ in children and adults, e.g., initial short acting beta agonist 400 microg in adults but in children is 200 microg increasing to 1,000 microg
Second line treatment	Same medications used (i.e., intravenous magnesium, salbutamol and aminophylline)	No major differences at this level

## Author Contributions

All authors listed have made a substantial, direct and intellectual contribution to the work, and approved it for publication.

### Conflict of Interest Statement

The authors declare that the research was conducted in the absence of any commercial or financial relationships that could be construed as a potential conflict of interest.
